# The dynamic landscape of gene regulation during *Bombyx mori* oogenesis

**DOI:** 10.1186/s12864-017-4123-6

**Published:** 2017-09-11

**Authors:** Qiang Zhang, Wei Sun, Bang-Yong Sun, Yang Xiao, Ze Zhang

**Affiliations:** 1 Laboratory of Evolutionary and Functional Genomics, School of Life Sciences, Chongqing University, Chongqing, 400044 China; 2 Sericulture & Agri-food Research Institute, Guangdong Academy of Agriculture Science, Guangzhou, 510640 China; 3 Laboratory of Evolutionary and Functional Genomics, School of Life Sciences, Chongqing University, Chongqing, 401331 China

**Keywords:** *Bombyx mori*, Oogenesis, Gene regulation, Ecdysteroid biosynthesis, Co-expression network, Transcription factors

## Abstract

**Background:**

Oogenesis in the domestic silkworm (*Bombyx mori*) is a complex process involving previtellogenesis, vitellogenesis and choriogenesis. During this process, follicles show drastic morphological and physiological changes. However, the genome-wide regulatory profiles of gene expression during oogenesis remain to be determined.

**Results:**

In this study, we obtained time-series transcriptome data and used these data to reveal the dynamic landscape of gene regulation during oogenesis. A total of 1932 genes were identified to be differentially expressed among different stages, most of which occurred during the transition from late vitellogenesis to early choriogenesis. Using weighted gene co-expression network analysis, we identified six stage-specific gene modules that correspond to multiple regulatory pathways. Strikingly, the biosynthesis pathway of the molting hormone 20-hydroxyecdysone (20E) was enriched in one of the modules. Further analysis showed that the ecdysteroid 20-hydroxylase gene (*CYP314A1*) of steroidgenesis genes was mainly expressed in previtellogenesis and early vitellogenesis. However, the 20E–inactivated genes, particularly the ecdysteroid 26-hydroxylase encoding gene (*Cyp18a1*), were highly expressed in late vitellogenesis. These distinct expression patterns between 20E synthesis and catabolism-related genes might ensure the rapid decline of the hormone titer at the transition point from vitellogenesis to choriogenesis. In addition, we compared landscapes of gene regulation between silkworm (Lepidoptera) and fruit fly (Diptera) oogeneses. Our results show that there is some consensus in the modules of gene co-expression during oogenesis in these insects.

**Conclusions:**

The data presented in this study provide new insights into the regulatory mechanisms underlying oogenesis in insects with polytrophic meroistic ovaries. The results also provide clues for further investigating the roles of epigenetic reconfiguration and circadian rhythm in insect oogenesis.

**Electronic supplementary material:**

The online version of this article (10.1186/s12864-017-4123-6) contains supplementary material, which is available to authorized users.

## Background

In eukaryotes, differentially fine regulation of various pathways at the level of transcription determines correct ontogenesis. Recently, relatively simple processes such as the oogenesis of the domestic silkworm *Bombyx mori* (Lepidoptera) and that of the fruit fly *Drosophila melanogaster* (Diptera) have been widely used to reveal the molecular mechanisms of developmental regulation [1]. Consequently, much research progress has been made toward understanding the changes in gene expression and the role of hormones in insect oogenesis [1–3].

The domestic silkworm *B. mori* and the fruit fly *D. melanogaster* have similar ovary structures, that is, polytrophic meroistic ovaries, which represent one type of insect ovaries. Oogenesis in polytrophic meroistic ovaries can sequentially be subdivided into three developmental periods: previtellogenesis, vitellogenesis and choriogenesis. Previous studies have suggested that the rise in the molting hormone 20-hydroxyecdysone (20E) titer in early pupae could trigger previtellogenic development and vitellogenesis in the domestic silkworm [3, 4]. The cause is the induction, by 20E, of the expression of a conserved cascade of regulatory genes such as nuclear receptors *E75*, *HR3*, and *Br-C* [3]. However, the transition from vitellogenesis to choriogenesis requires another cascade of gene expression that is controlled by a decline in the 20E titer. While *Drosophila* oogenesis is regulated by both juvenile hormone (JH) and 20E [5], it is found that the oogenesis of the domestic silkworm depends only on 20E signaling [6]. This finding suggests a conserved role for 20E signaling in the oogenesis of both the domestic silkworm and fruit fly. Thus, the role of 20E in insect oogenesis has become a focus of research. A recent study used genetic mosaic analysis to screen putative 20E–responsive genes for novel roles in the control of the earliest stages of *Drosophila* oogenesis [7].

**Fig. 1:**
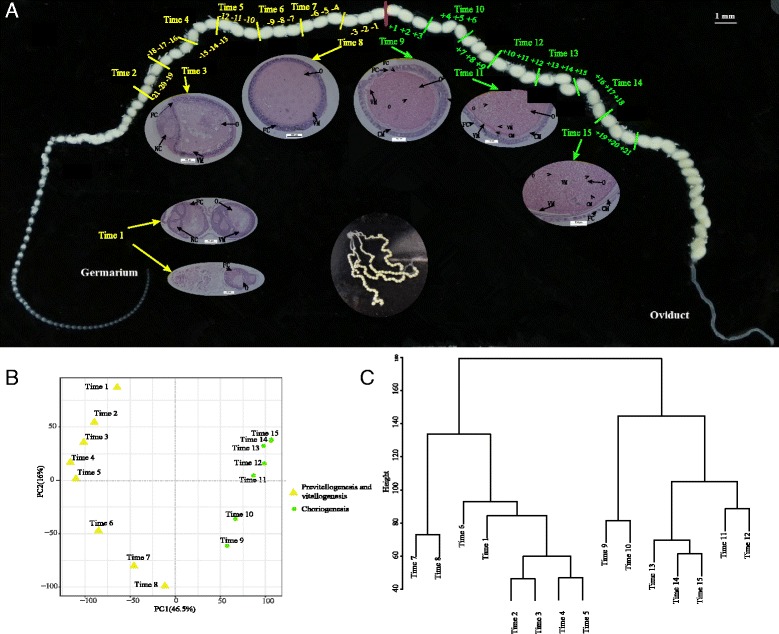
Dissection, PCA and clustering of samples in *B. mori* oogenesis. **a** Dissection of sequenced samples in a single ovariole of the day 7 pupa. The central elliptical figure shows the single ovary of the day 7 pupa containing four ovarioles. Each ovariole was divided into 15 samples, based on the transition point from vitellogenesis to choriogenesis, represented by a short red line. The samples of previtellogenesis and vitellogenesis are marked in yellow. The choriogenesis samples are marked in green. The microstructures at Time 1, Time 3, Time 8, Time 9, Time 11 and Time 15 were obtained. O and NC represent oocyte and nurse cells, respectively. FC represents follicular cells. CM represents chorion membrane. All microstructures were scaled to 100 μm. **b** Principal component analysis results of total 15 samples based on the expression profiles. Different colors indicate various groups. **c** Clustering dendrogram of samples based on their average Euclidean distance

Although the roles of several transcription factors in the oogeneses of the domestic silkworm and fruit fly have been well known, the dynamic landscape of gene regulation at the genome scale during the oogenesis of polytrophic meroistic ovaries remains to be determined. In the domestic silkworm, each ovary consists of 4 ovarioles, and each ovariole can contain up to 75–80 follicles or eggs, which are organized into a single array (Fig. 1a). Each developing follicle is separated by 2–2.5 h of developmental time from its immediate neighbor [3]. This unique feature of the system makes it possible to simultaneously isolate all stages of follicle development from a single animal for physiological, biochemical and gene expression studies. Thus, the developing ovariole of the domestic silkworm provides an excellent model for studies on the changes in gene expression during the execution of long-term developmental programs [3, 8]. To exploit this advantage, in this study we obtained multiple transcriptomes at different time points of ovariole development by next-generation RNA sequencing. The goal of the study was to determine the dynamic landscape of gene regulation during domestic silkworm oogenesis by analyzing time-series transcriptome data.

## Results

### Drastic morphological changes during oogenesis

In polytrophic meroistic ovarioles, the germarium is the area at the tip of the ovarioles where egg formation is initiated, containing germ line stem cells, somatic stem cells, and their niches [9]. The microscopic structure of the *B. mori* germarium showed that only one oocyte at the center was enveloped by follicle cells in disordered fashion during previtellogenesis (Time 1 in Fig. 1a). Proceeding to the vitellogenesis stage (Time 3 in Fig. 1a), the follicles were entirely covered by the vitelline membrane. Meanwhile, the interconnected nurse cells began to shrink and gradually degenerated. In contrast, follicle cells close to the oocyte-nurse cell interface commenced to migrate centripetally between the oocyte and the nurse cells, and the oocyte grew and then completely occupied the follicular volume during late vitellogenesis (Time 8 in Fig. 1a). Furthermore, a loose chorion layer formed at the onset of choriogenesis (Time 9 in Fig. 1a). The translucent chorion layer then became dense, and cavities formed between the vitelline membrane and the follicle cells layer during middle choriogenesis (Time 13 in Fig. 1a). During late choriogenesis, the nuclei of follicle cells became sparse and decreased in number during late choriogenesis (Time 15 in Fig. 1a).

### Transcriptome sequencing and confirmation of developmental transition

We investigated the profiles of gene expression during follicle maturation in *B. mori* by analyzing RNA-seq data of transcriptome at various development stages. The overall mapping rates for every sample ranged from approximately 60% to 80% (Additional file 1), and the expression profiles of typical genes validated by qRT-PCR were consistent with the transcriptomic changes of RNA-seq data (Additional file 2). Principal component analysis (PCA) results showed that all samples were divided into two distinct groups (Fig. 1b), separated at the transition from vitellogenesis to choriogenesis. Hierarchical clustering also divided the samples into two clusters (Fig. 1c). In addition, the expression patterns of marker genes showed distinct, stage-specific features. Genes encoding Foxo were exclusively expressed during vitellogenesis, and the members of the *chorion* family were divided into early, middle and late subgroups during choriogenesis according to the temporal expression patterns of typical chorion genes, like the early chorion genes (*ErB.2* and *ErB.4*), the middle chorion genes (*BmCho-1*) and the late chorion genes (*HcB 12*) (Additional file 3). Taken together, five groups could be referred to different oogenesis stages. The first (Time 1 to Time 6) and second groups (Time 7 and Time 8) included the stages from previtellogenesis to middle vitellogenesis and late vitellogenesis. The third (Time 9 and Time 10), fourth (Time 11 and Time 12) and fifth groups (Time 13 to Time 15) represented the early, middle and late choriogenesis stages, respectively. These results are consistent with the morphological and physiological changes that occur during oogenesis.

### Drastic changes in gene expression at developmental transition points

**Fig. 2:**
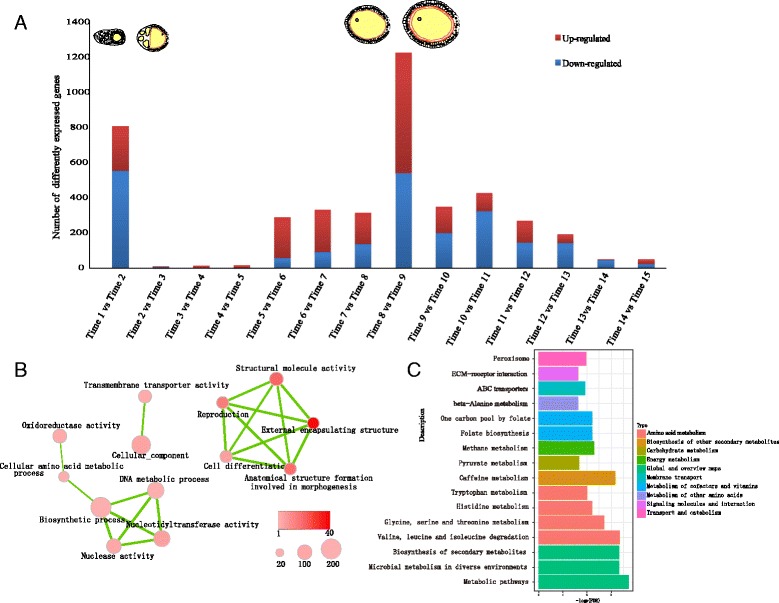
Statistics of total differentially expressed genes. **a** Numbers of total differentially expressed genes between adjacent samples. The numbers of up-regulated and down-regulated genes are represented in red and blue, respectively. The graphics at the top show the main changes of cell types and structure of follicles during the oogenesis, which is the abstract rendering of the microstructures in Fig.1A. The outer cells represent the follicle epithelial cells. The yellow parts represent the oocyte and the nurse cells. The blue circles represent the nuclei of follicle epithelial cells and oocytes. The brown and red lines represent the vitelline membrane and chorion layer. **b** Enrichment map for significantly enriched GO terms. The level of overlap between GO enriched terms is indicated by the thickness of the edge between them. The size of the node indicates the size for the term, and the gene group for which the term was found to be significant (FDR < 0.05, Overlap coefficient cut-off >0.25); the color of the node indicates the absolute value of the base 10 logarithm of the FDR. **c** Enrichment map for significantly enriched KEGG pathways

To identify the differentially expressed genes during follicle maturation, we compared the gene expression levels between adjacent stages. A total of 1932 unique genes were identified to be differentially expressed between all consecutive stages. Similarly to the observed morphological changes, differential expressions of most genes mainly occurred at the two transition points from previtellogenesis to vitellogenesis and from vitellogenesis to choriogenesis (Fig. 2a). The functional GO terms of those genes were mainly enriched in reproduction, cell differentiation, morphogenesis and transmembrane transport (Fig. 2b), highlighting the typical biological progress of germ cell differentiation. Using KEGG enrichment analysis, we found that differently expressed genes were enriched in the classifications of metabolism of energy and cofactors, and environmental information processing, including ABC transporters and ECM-receptor interaction pathways (Fig. 2c). Additionally, 686 and 542 genes were up-regulated and down-regulated at the transition between late vitellogenesis (Time 8 in Fig. 1a) and early choriogenesis (Time 9 in Fig. 1a), respectively. The down-regulated genes are specifically involved in enzyme regulator activity, isomerase activity, proteinaceous extracellular matrix, and the lipid metabolic process. Conversely, the genes involved in cellular localization, death, cell localization, macromolecule localization and regulation of biological quality were specifically up-regulated (Additional file 4), showing drastic changes in biological progress around the transition from vitellogenesis to choriogenesis.

**Fig. 3:**
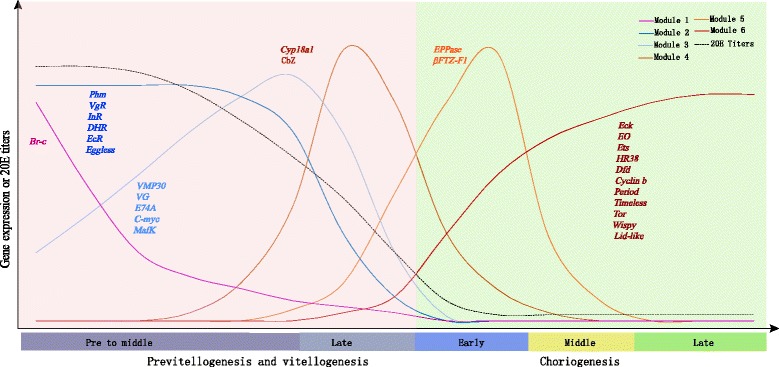
Schematic diagram of co-expressed modules fluctuating over time and 20E titer. The total development time shown on the x-axis is divided into five stages based on the expression profiles of marker genes and morphological changes. The y-axis represents the expression of genes or the titer changes in 20E. The various solid-colored lines represent the expression profiles of the module eigengenes of the same color. The dotted line indicates the titer of 20E. The typical maker genes are listed around the colored lines of the module eigengenes. The explanation of gene identities is in the Additional file 12

**Fig. 4:**
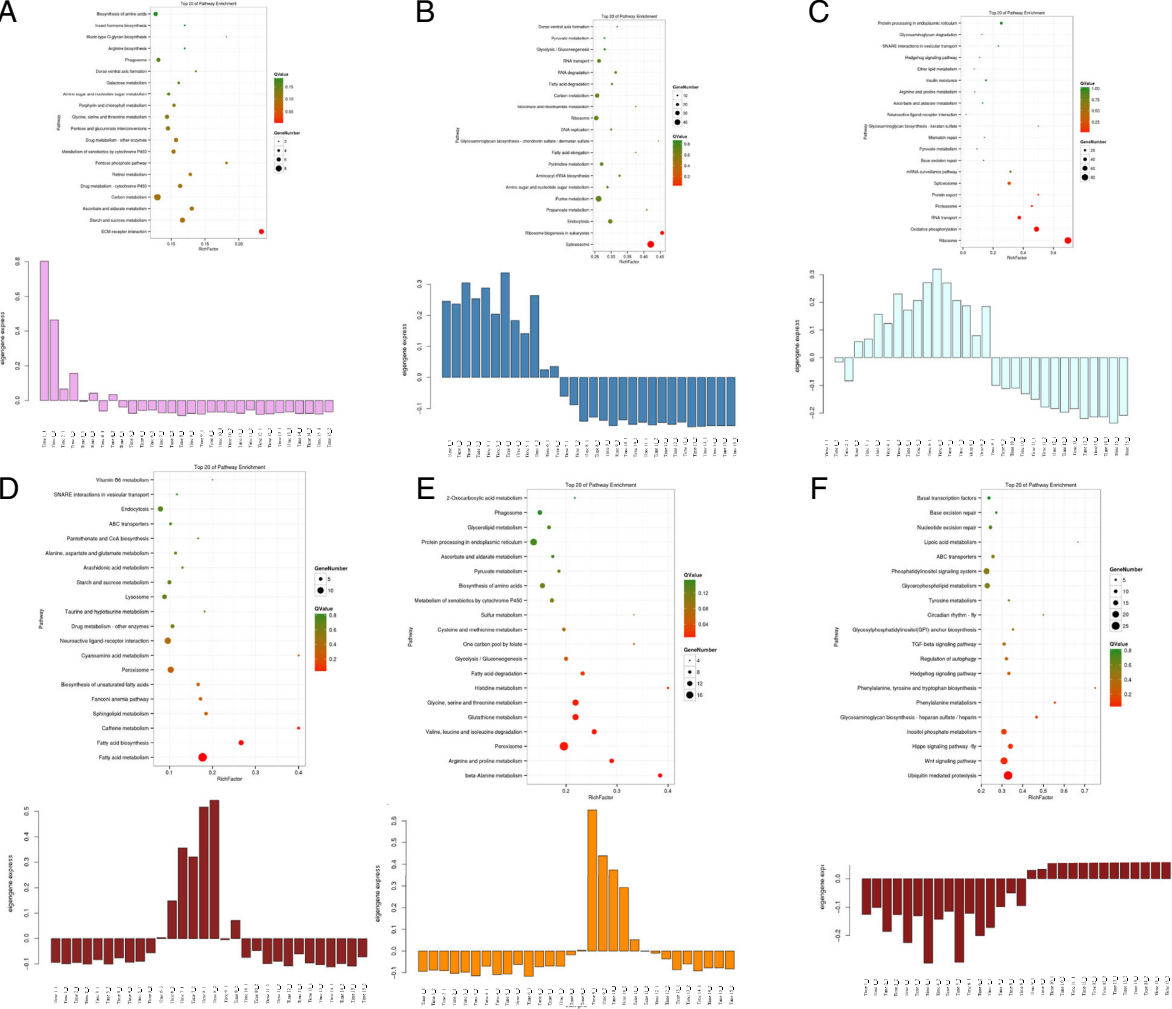
KEGG enrichment results of co-expressed modules. **a**-**f** Each figure contains the KEGG enrichment result and the expression histogram of module eigengenes from Module 1 to Module 6. Q-value means FDR using Benjamini and Hochberg correction method

We also identified six modules corresponding to distinct development stages during oogenesis (Fig. 3). Each module was enriched in specific functional classification and pathway. For example, the genes in Module 1 were exclusively and highly expressed at the onset of oogenesis (Time 1 and Time 2 in Fig. 1a) and significantly enriched in immune system process (Additional file 5). Furthermore, the dorso-ventral axis formation and insect hormone biosynthesis pathways were highlighted as key regulatory pathways (*p*-value <0.05, FDR < 0.15) (Fig. 4a). In Module 2, the genes were significantly enriched in methyltransferase activity, ncRNA metabolic process and spliceosome pathway (Fig. 4b), suggesting specific regulatory processes. In Module 3, the genes were significantly enriched in biosynthetic process, transport, localization, modification process and oxidative phosphorylation pathway, indicating the main preparation and protein storage phases (Fig. 4c). Module 4 mainly included genes involved in cell adhesion, transmembrane transporter activity and oxidoreductase activity and pathways of fatty acid metabolism, fatty acid biosynthesis and biosynthesis of unsaturated fatty acids (Fig. 4d), suggesting that late vitellogenesis was an important period for energy storage. Moreover, the genes in Module 5 were specifically expressed in early choriogenesis and were enriched in the GO categories of reproduction and cytoskeleton and the fatty acid degradation pathway (Fig. 4e). Module 6 nearly covered the entire choriogenesis period, mainly including the genes involved in the Wnt, Hippo, Hedgehog, TGF-beta signaling pathways as well as autophagy regulation (Fig. 4f); biological processes such as cell communication, signal transduction, stimulus response, autophagy, cell proliferation, cell division, mitotic cell cycling and cell death were also enriched in this module.

To identify the key regulatory factors in each module, we first identified 761 transcription factors (TFs) and 90 cofactor genes by genome-wide screening. Total TFs were classified into 7 superclasses and 60 TF families based on the characteristics of their DNA-binding domains (Additional file 6), including the Forkhead and HMG_box families [10, 11], which are known to play a regulatory role in oogenesis. In Module 1, many key factors’ responses to 20E signaling were observed, for example, *Br-c*, *Eip78C* and *Notch* [12, 13]. For Module 2, *SIX4*, *HMGA* and *eggless* in particular were also highly expressed throughout nearly the entire vitellogenesis period, suggesting that TFs and epigenetic factors might play a joint role in a tight regulatory system. Module 3 included *MafK*, *E74A* and *c-myc*, which have been reported to play a role in *D. melanogaster* oogenesis [14]. *CbZ* and *FTZ-F1* in Modules 4 and 5, respectively, were previously identified as the main regulatory genes for choriogenesis [15, 16]. The diapause-associated genes *timeless*, *Ets* and *Tret1* were mainly expressed in Module 6, suggesting that choriogenesis may be the critical period for egg diapause [17].

### Hormone regulation of *B. mori* oogenesis

**Fig. 5:**
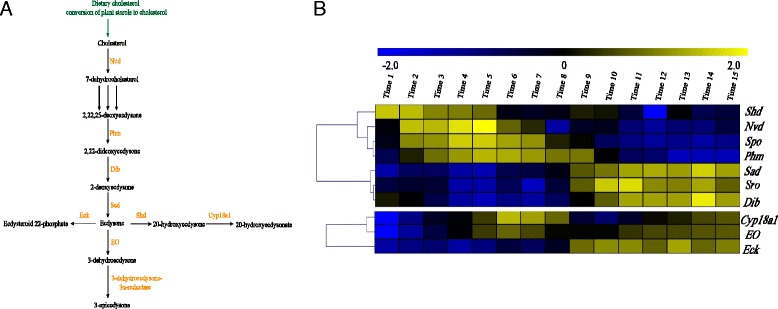
Schematic diagram and expression profiles of ecdysteroid synthesis and metabolism pathways. **a** Schematic diagram of ecdysone synthesis and metabolism pathways in *B. mori* and *D. melanogaster*. The pathways from BioCyc Database Collection were modified for *B. mori* and *D. melanogaster*. Ecdysones were converted from dietary cholesterol, marked in green. A series of well-known synthesis and metabolism enzymes are marked in orange beside catalysates. **b** The expression changes of seven synthesis enzymes of ecdysteroids and three metabolism enzymes

Pathway analysis showed that the insect hormone biosynthesis pathways were enriched during the initial stage of oogenesis. Furthermore, nearly every module harbored many TF responses to 20E signaling, suggesting that 20E signaling plays an important regulatory function in oogenesis. To confirm the regulatory function of 20E in oogenesis, an experiment in which exogenous 20E and 20E agonist tebufenozide (RH-5992) were injected into ligated pupa abdomens was performed. The ovaries of individuals treated by being injected with exogenous 20E developed normally, whereas the development of the ovaries in control individuals was arrested (Additional file 7A, B), indicating that 20E is essential for the first transition from previtellogenesis to vitellogenesis. However, although the ovaries in individuals injected with RH-5992 could develop, the development of follicles was soon blocked [18]. Thus, the second transition from vitellogenesis to choriogenesis could not be observed (Fig. 5c). These observations suggested that 20E was the key regulator for the formation of the two transitions during oogenesis. Furthermore, we examined the changes in the 20E titer in female ovarioles during pupal-adult development. As shown in Fig. 3, the 20E titer reached its maximum level during the early stage of vitellogenesis, then decreased drastically before the transition from vitellogenesis to choriogenesis. The changes in the 20E titer were consistent with the expression profiles of the genes involved in 20E synthesis and metabolism (Fig. 5a). The expression levels of ecdysteroidogenic enzyme genes showed distinct scheduling (Fig. 5b). The gene *Shd* (*CYP314A1*) encoding *ecdysteroid 20-hydroxylase*, the key enzyme that transforms ecdysone into 20E, was highly expressed at the initial stage of oogenesis. This finding corresponded with the high 20E titer (Fig. 3). In addition, the expression level of the gene *CYP18A1* regulating steroid hormone inactivation specifically increased before the transition point from vitellogenesis to choriogenesis (Fig. 5b, Additional file 2), suggesting that this metabolic enzyme may result in a decrease in 20E during this transition. Furthermore, several intermediate receptors (*EcR*, *DHR* and *InR*) for hormone signaling exhibited stage-specific expression patterns during the early stage of oogenesis (Fig. 3), suggesting that this stage is highly important for the functions of hormone regulatory signaling. Thus, we performed injection experiments involving several inhibitors of the insulin signaling pathway. It was observed that the development of follicles was clearly delayed and the follicles showed abnormalities (Additional file 7), and the expressions of marker genes, like *Cyp18a1* and early chorion gene, were up-regulated at choriogenic stages. Probably, insulin signaling may be related to the normal progression of oogenesis.

### Consensus oogenesis regulatory network between *B. mori* and *D. melanogaster*

Because *B. mori* and *D. melanogaster* have similar ovary structures and the data on *D. melanogaster* oogenesis have been made available, we performed a comparative analysis of the oogenesis regulatory network. Similarly, the genes encoding the york proteins and vitelline membrane proteins were specifically expressed during the early period of oogenesis from stage 1 to stage 10 of *D. melanogaster* oogenesis, while genes encoding chorion proteins were mainly expressed from stage 11 to stage 14 (Additional file 8A). Thus, *D. melanogaster* oogenesis was also divided into two distinct phases, previtellogenesis with vitellogenesis and choriogenesis. The *shd* gene was expressed during previtellogenesis and vitellogenesis before the transition, while the expression level of *Cyp18a1* increased during the late stages (from stages 11 to 13, Additional file 8B), suggesting that a low 20E titer was required for the transition from vitellogenesis to choriogenesis. This result is similar to that obtained for *B. mori*. In addition, we identified four consensus modules that harbor 176 TFs and chromatin remodeling factors. In these modules, 233 genes were known to play an important role in oogenesis based on the mutation phenotypes in *D. melanogaster* (Additional file 9). Strikingly, the ecdysteroid synthesis-related genes, such as *nvd*, *dib*, *sro*, *phm* and *sad*, and the known factors of oogenesis responding to 20E signaling, such as *Pxt* and *Iswi*, are enriched in the consensus modules [19, 20]. This indicated that the ecdysteroid synthesis and some hormone signaling pathways in ovaries were conserved in both *B. mori* and *D. melanogaster* [19, 21].

## Discussion

Each follicle is a functional unit containing a single oocyte in insects, which is essential for the generation of healthy oocytes. However, the mechanisms that regulate the oogenesis have not been well known. Two typical morphological changes occur during the oogenesis of insects, the accumulation of the yolk protein and the formation of chorion, resulting in two main transitions from previtellogenesis to vitellogenesis and from vitellogenesis to choriogenesis. Our time-series transcriptome data obtained during silkworm oogenesis do reflect that the molecular process of follicle development is much different between vitellogenesis and choriogenesis. Furthermore, it can be inferred from our results that the synthesis of amino acids and ribosomes in vitellogenesis occurs in preparation for macromolecular modification in choriogenesis (Additional file 5). It should be realized that the developing follicles consist of two types of cells: germline cells (oocyte/nurse cells) and somatic cells (follicular epithelium). Due to the small size of follicles, cells of different types were difficult to dissect, respectively. This limited the investigation of separate developmental dynamics of different cell types. However, due to the molecular substance and signal exchange between different cell types, the homogenized developing follicles could be considered as a functional unit. In addition, direct identification of maternal genes was difficult using this data, because of the lack of the cell type information and the change of protein levels. To a certain extent, morphologic observation could supplement the missing information for dynamics of different cell types. For example, the genes expressed continually during choriogenesis were likely to be maternal genes, because at that time the follicle cells gradually experienced apoptosis and oocyte was uniquely reserved in the mature egg [22]. Previous studies have also suggested that maternal mRNAs are mainly polyadenylated during late oogenesis in *D. melanogaster* and that regulation of poly (A) tails in oocytes shapes the translatomic landscape of embryos [23]. Indeed, we found that *Wispy*, a typical marker gene for studying maternal factors, was highly expressed during choriogenesis. This result is consistent with the accumulation of maternal mRNAs during *B. mori* choriogenesis. In addition, we found that several signaling pathways important for embryogenesis were also enriched in choriogenesis (Fig. 4f). This result implies that these molecular processes may occur in preparation for late embryo development. Energy metabolism is generally believed to be important for molecular processes. Our data showed that fatty acid biosynthesis and unsaturated fatty acid biosynthesis mainly occurred during the late stage of vitellogenesis, whereas their metabolism occurred during early stage of choriogenesis (Fig. 4d, e). This finding may reflect that fatty acid metabolism provides the energy needed for the transition from vitellogenesis to choriogenesis.

Recent studies have shown that various hormones may be responsible for the expression regulation of the genes involved in the two transitions during oogenesis in insects. In *Tribolium castaneum, Blattella germanica* and *Locusta migratoria*, the initiation of oogenesis depends on JH [24–26]. In *D. melanogaster*, the appropriate balance between JH and 20E is crucial for the normal progress of oogenesis [27]. Our ligation experiments on prepupae suggested that 20E played a central role in *B. mori* oogenesis. Thus, a high 20E titer is essential for the transition from previtellogenesis to vitellogenesis, whereas the decline in the 20E titer results in the transition from vitellogenesis to choriogenesis. This result is in accord with the 20E titer changes we observed during various stages of oogenesis. Furthermore, we investigated the expression patterns of 20E synthesis- and metabolism**-**related genes. Our results showed that the stage-specific expressions of these related genes reflected the 20E titer changes at the transition point during oogenesis. In addition, we compared the regulatory network of genes between *B. mori* oogenesis and *D. melanogaster* oogenesis because these two insects have similar ovary structures. The source of ecdysone controlling oogenesis in *B. mori* ovaries is different from that in *D. melanogaster* ovaries. The ecdysteroid 20-hydroxylaseare-encoding gene (*Shd*) was expressed at the onset of oogenesis in *B. mori*. At this stage, ecdysteroid is not completely synthesized in the ovaries, which is induced by 20E [28, 29]. Thus, the ecdysone controlling oogenesis is mainly released from the prothoracic glands, indicating that the ecdysteroids produced by the ovaries have no autocrine/paracrine or endocrine function to regulate the progression of oogenesis in the silkworm. However, in *D. melanogaster*, the *Shd* gene was expressed until late vitellogenesis, in accord with sequential expressions of other ecdysteroid synthesis-related genes in follicles. This result implies that the ecdysone produced in ovarian follicular cells, which is stimulated by JH, may play a paracrine or autocrine regulatory role in oogenesis [30–33]. Although *B. mori* oogenesis is only related to 20E and *D. melanogaster* oogenesis is controlled by both 20E and JH, the mechanisms of ecdysteroid synthesis and metabolism in the two insects are similar to each other. For the two insects, increase and decrease in the 20E titer are required for the transitions from previtellogenesis to vitellogenesis and from vitellogenesis to choriogenesis, respectively. This finding suggests that the gene regulatory subnetwork stimulated by 20E in ovaries is evolutionarily conserved. In addition to finding consistency in 20E regulation, the different subnetworks may shed some light on the unique characteristics of hormonal regulation of these two insects oogenesis.

**Fig. 6:**
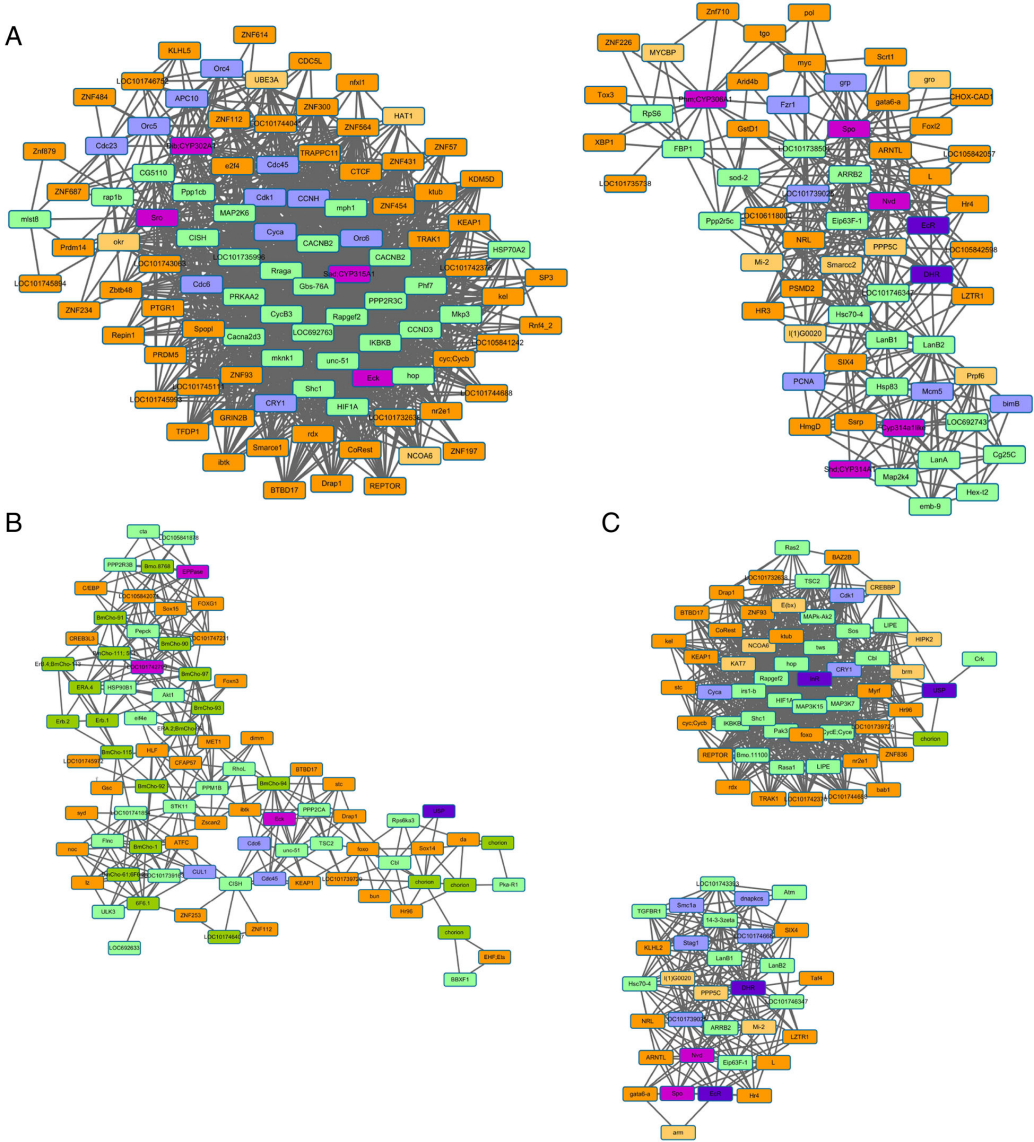
Co-expressed sub-network of pathways associated with ecdysteroid, insulin signaling and circadian rhythm during oogenesis. **a** The sub-network of ecdysteroid biosynthesis. **b** The sub-network of chorion regulation. **c** The sub-network of typical hormone receptor. The pathways, including insect hormone biosynthesis, insulin signaling pathway, mTOR signaling pathway, PI3K-Akt signaling pathway, MAPK signaling pathway, FoxO signaling pathway and circadian rhythm pathway, were investigated with the co-expressed connections of putative TFs and cofactors. The ecdysteroid synthesis and metabolism enzymes are marked in purple. The receptors are shown in dark purple. The TFs and cofactors are represented in dark orange and light orange, respectively. Genes of the circadian rhythm pathway and insulin pathways are marked in light blue and light green, respectively. The chorion proteins family in (**b**) are represented in green. The explanation of gene identities is in the Additional file 12

Previous studies put forward a model to address the question of how promoter architecture facilitates developmentally accurate chorion gene regulation, which described both cis-elements and their corresponding transcription factors (TFs) were important for the developmental regulation of chorion genes [34]. The BmC/EBP transcription factor was considered both as an activator and a repressor during choriogenesis in *B. mori*. BmGATAβ-binding was possibly related to early repression of early and middle chorion gene expression and late activation of late chorion gene pairs. BmHMGA-factor-binding to middle chorion gene promoters recruits other TFs and causes bending of the promoter [1, 35]. The expressions of chorion genes were correlated with the expression of these factors of oogenesis. Furthermore, other factors, like *CbZ* and *FTZ-F1*, were expressed around the transition point from vitellogenesis to choriogenesis. Thus, we identified the TFs and cofactors co-expressed with chorion genes and the known regulators in each module. Many zinc finger proteins, like *ush* and fork head domain transcription factors were in the same module with the known factors (Additional file 10A). The subnetwork of chorion and their co-expressed TFs also gave a hint to find the putative regulators, like Sox15, FOXG1 and HLF (Fig. 6b). In this study, a co-expression analysis of the genes involved in 20E and insulin signaling regulation was also performed. Similar to the previous findings that *USP* and *FoxO* have physical interactions [36], we found that *InR*, *FoxO*, *USP* and *HR96* are co-expressed during oogenesis (Fig. 6c). An experiment involving the injection of insulin signaling inhibitors also confirmed that insulin is an important factor for maintaining the normal development of follicles. Although the morphological changes are similar by RH-5992 and inhibitors of insulin signaling pathway, follicles developed slowly and the yolk accumulation decreased, the inhibitory effects were different. RH-5992 blocked the 20E pathways, and the developmental arrest by RH-5992 cannot be rescued by culturing the follicles in vitro. However, the developmental arrest under small doses of inhibitors of insulin signaling pathway can be rescued. Indeed, we observed that only the duration of pupa kept longer while the ovary developed normally (data not shown). Whether insulin signaling pathway is related to oogenesis or whether there are dosage effects of insulin signaling inhibitors on oogenesis remains to be further investigated in future. In addition, recent studies have shown that the central circadian clock genes *period* and *timeless*, which do not encode actual TFs but encode regulatory proteins that modulate transcription, are rhythmically expressed in the PG and play a role in controlling development rhythms. Physical interactions among *CDK8*, *CyclinC*, *EcR*, and *USP* have also been detected [37]. Similar interactions between JH signaling and circadian clock genes that act at the organ-autonomous level in *Pyrrhocoris apterus* and *Aedes aegypti* have been well documented [38, 39]. Thus, the gene expression levels of a certain set of genes in the follicles might be transcriptionally regulated under the control of the central clock network, which may elucidate the autonomous regulatory mechanism of oogenesis in *B. mori*. Our results showed that ecdysteroid biosynthesis enzymes such as *Sro*, *Dib* and *Sad* and the circadian clock components *Cyca* and *Cycb* were co-expressed during oogenesis (Fig. 6a). This finding suggests that the autonomous temporal expression of biosynthesis enzymes we observed may be the result of strictly circadian regulation. Certain circadian rhythm genes, such as *CRY1* and *CycE*, are also co-expressed with *USP* and *InR*, implying that circadian genes may function downstream of multiple regulatory hormone signaling.

Additionally, histone methylation is likely the most extensively studied epigenetic modification during oogenesis. In *D. melanogaster*, *Piwi* negatively regulates Polycomb group proteins mediated by histone 3 lysine 27 trimethylation (H3K27m3) in maintaining germline stem cells and oogenesis. Indeed, our data showed that the histone methylation-related gene *eggless* was highly expressed during vitellogenesis, whereas histone demethylase (lysine-specific demethylase *lid*) was highly expressed during choriogenesis (Fig. 3, Additional file 10B), suggesting a molecular switch in the dynamics of histone lysine methylation at the transition from vitellogenesis to choriogenesis. Further dedicated experiments are required to determine the contribution of epigenetic reconfiguration to the regulatory reorganization of the transcriptional program with 20E [40, 41].

## Conclusions

In summary, our findings reveal the important role of 20E in inducing the related cascades of molecular processes during *B. mori* oogenesis and accordingly elucidate the dynamic landscape of gene regulation. These results will expand our understanding of the details of insect oogenesis. Strikingly, the data presented in this study also provide clues for further investigating the roles of epigenetic reconfiguration and circadian rhythm in insect oogenesis.

## Methods

### Experimental sample collection and next-generation RNA sequencing

The polyvoltine strain 305 of the domestic silkworm was reared on fresh mulberry leaves at 25 °C under a 12 h-light:12 h-dark photoperiod. The ovarioles in pupae on day 7 (P7D) were dissected in physiological saline, and the dissected ovarioles were then infiltrated with 90% glycerol for 10 min at room temperature to distinguish the −1/+1 transition point from vitellogenesis to choriogenesis. Every three sequential follicles from 3 individuals were homogenized as one time sample from the transition point to both sides, the last part of ovarioles was discarded and the retained follicles at the onset were pooled as the first sample. In total, 15 samples labeled Time 1 to Time 15, from previtellogenesis to choriogenesis, were obtained (Fig. 1a). Total RNA was extracted from two biological repetitions using TRIzol (Invitrogen, USA) according to the manufacturer’s instructions and treated with DNase I. The mRNA was purified, and used to library construction. The library was sequenced using an Illumina HiSeq™ 2000 platform with a 125 paired-end module at Novogene (Beijing, China).

### Assembly of transcriptome and annotation

The raw reads were trimmed and filtered using Trimmomatic software (v0.33) to remove adapters and low-quality bases. The clean reads were then realigned and assembled into transcripts using TopHat (v2.0.13) and Cufflinks (v2.2.1) with the *B. mori* genome from SilkDBv2.0 as a reference [42, 43]. Cuffmerge and Cuffnorm were used to calculate expression at the gene and transcript levels in fragments per kilobase of exon model per million mapped fragments (FPKM) and reads per kilobase of exon model per million mapped fragments (RPKM). All assembled transcript sequences were annotated by the UniProt databases, NCBI NR databases and Kyoto Encyclopedia of Genes and Genomes (KEGG) database using BLAST with an E-value threshold of 10^−5^; the Gene Ontology (GO) terms were inferred from the best hit using Blast2GO [44]. The replicates of all samples were analyzed by the Pearson correlation method. Principal component analysis (PCA) and hierarchical clustering were performed using the sample profiles to obtain an overview of the sample relationships in R (v3.3.1).

### Identification of transcription factors, cofactors and typical gene families

All protein sequences predicted by TransDecoder were searched against the Pfam database by hmmscan in the HMMER package (v3.1b1), with 10^−4^ as the E-value [45]. Then, sequences with hidden Markov model (HMM) profiles from the Animal Transcription Factor DataBase (AnimalTFDB) and REGULATOR database were identified as TFs, except for the known receptors, enzymes and proteins associated with histone and transposon [46, 47]. The genes with GO terms related to chromatin remodeling were categorized as cofactors. The typical gene families involved in follicle maturation, including nutrition proteins (*30 K* and *ESP*) and structure proteins (*chorion* and vitelline membrane protein), were also identified.

### Differential expression analysis and construction of gene co-expression network

The gene expression profiles of adjacent stages were compared by DEseq2 [48]. The genes that had a two-fold difference in expression level between two stages and a false discovery rate (FDR) less than 0.05 were identified as differentially expressed genes. A co-expression network was constructed using the weighted gene co-expression network analysis (WGCNA) package [49]. Total gene profiles were imported to generate the modules with a soft threshold parameter β = 10, and modules were merged when their eigengene correlation was higher than 0.7. The 20E titers of different follicles were added as phenotypic features to evaluate the modules’ relationships with regulatory hormone. After subjecting the data to strict quality control assessments, the top six modules with good consistency were selected for downstream analysis. The co-expressed interactions among regulators and genes involved in signaling pathways were visualized using Cytoscape (3.2) [50]. The GO function and pathway enrichment analyses were tested using the FDR (Benjamini and Hochberg correction method, FDR < 0.05). Only genes having gene symbols in top terms are shown in Additional file 5.

### Consensus network construction of cross-species analysis

The oogenesis transcriptomes from *D. melanogaster* at different development stages were collected from the NCBI Sequence Read Archive (SRA) and Gene Expression Omnibus (GEO) (Accession: PRJNA257462, PRJNA326885, GSE83616) [23, 51]. We obtain the RPKM expressions of total coding genes in each samples using the same procedure in *B. mori*. We then detected the consensus modules between *D. melanogaster* and *B. mori* oogenesis using a procedure similar to that reported in previous studies [52]. The powers for transforming the co-expression matrices were scaled by topology model fit value. Module preservation statistical tests were used to analyze module preservation across different datasets. We calculated 200 permutations of the preservation statistics and generated a Z-summary value by averaging them. The Z-summary indicates whether a module is strongly preserved (Z-summary score > 10), moderately preserved (5 < Z-summary score < 10), or not preserved (Z-summary score < 5). To visualize module structures, we extracted the genes with the highest module membership and the strongest gene-gene connections among these from the signed topology overlay matrix to visualize the networks.

### Real-time quantitative PCR analysis

Ovary samples were dissected from *B. mori* as described above and stored at −80 °C. A TransZol Up Plus RNA Kit (Transgen, China) was used to isolate total RNA, and 1.5 μg of total RNA free of genomic DNA contamination was used to synthesize cDNA with oligo d (T) primers. The relative expression levels of 12 genes that serve as biomarkers for oogenesis and typical pathways, such as the ecdysteroid biosynthesis pathway and the insulin signaling pathway, were measured using a Bio-Rad CFX System (Bio-Rad, USA) with SYBR *Premix Ex Taq* II Supermix (Takara, Japan) according to the manufacturer’s instructions. PCR amplification was conducted under the following conditions: 95 °C for 30 s, followed by 40 cycles at 95 °C for 10 s and at 60 °C for 30 s. The gene expression levels were calculated by the 2^-△Ct^ method and normalized against an internal reference gene, *BmRpl3* (GeneBank accession: HQ615689). For each sample, three biological replicates were taken based on the average value, and the standard error was calculated. The primers used in this study were designed by Primer Premier 5 and are shown in Additional file 11

### Exogenous hormone and inhibitors in vivo injection

We made the ligation in the chest in the prepupa stage and then injected the exogenous 20E and 20E agonist tebufenozide (RH-5992) into the pupa abdomen on day 1 at a concentration of 2 μg/μl. The control groups were injected with 30% alcohol. The ovary was dissected from pupa on day 6. To examine the role of the insulin signaling pathway in follicle development, we used several inhibitors (LY294002, U0126 and Rapamycin, AbMole BioScience, USA) to block the pathway of insulin signaling. LY294002 is a highly selective inhibitor of phosphatidylinositol 3 (PI3) kinase. U0126 is a highly selective inhibitor of both MEK1 and MEK2, a type of MAPK/ERK kinase. Rapamycin is an inhibitor of mTOR. All inhibitors were used in previous researches of the ecdysone biosynthesis in the prothoracic gland [53–55]. The inhibitors were injected into the hemolymph of pupae on day 2 and day 4, respectively. The concentration of the injection was twice the dosage used in the prothoracic gland [53–55]. The control groups were injected with DMSO. The ovary was dissected to be observed 48 h after injection. Ovarioles of pupa on day 6 were cultured in Grace’s medium (Gibco) at 25 °C for 48 h in the absence or in continuous presence of inhibitors (LY294002, U0126 and Rapamycin), then dissected follicles at choriogenic stages (+1 − +6). The gene expression of typical genes (Cyp18a1 and Era) by qRT-PCR. The method of qRT-PCR was described above.

### Paraffin sectioning and 20E titer detection

Follicle samples of *B. mori* were collected as described above. The 20E titer at every developmental timepoint was detected by *B. mori* 20E ELISA Kit (Huijia BioScience, China) according to the accompanying manual. The same samples were fixed with 4% paraformaldehyde at 4 °C and then cut using the normal paraffin sectioning method and stained by the typical hematoxylin-eosin method.

## Additional files

Additional file 1: The statistics of sequencing samples. (XLS 29 kb)

Additional file 2: The quantitative PCR expression results of typical marker genes. The explanation of gene identities is in the Additional file 12. (PDF 444 kb)

Additional file 3: The expression profiles for typical protein families during oogenesis in *B. mori*. (PDF 1351 kb)

Additional file 4: The GO classifications of differentially expressed genes between Time 8 and Time 9. The up-regulated and down-regulated genes are marked in red and blue, respectively. The classifications of molecular function, cell component and biological process are represented in different colors. (PDF 600 kb)

Additional file 5: The key factors in co-expressed modules during oogenesis in *B. mori*. (XLS 27 kb)

Additional file 6: The statistics of transcription factors. (A) The classification of TFs. (B) TF families with the top 10 numbers. (PDF 301 kb)

Additional file 7: The morphological changes of ovaries following hormone and inhibitor treatments. (A-C) The morphological changes observed following the 20E, 30% alcohol and RH-5992 treatments, respectively. (D-G) The morphological changes in the DMSO control and various inhibitors of insulin pathways, LY294002, U0126 and Rapamycin, respectively. (H-I) The quantitative PCR expression results of typical marker genes. The explanation of gene identities is in the Additional file 12. (PDF 14262 kb)

Additional file 8: The expression profiles of typical genes during oogenesis in *D. melanogaster*. (A) The expression profiles for typical protein families during oogenesis in *D. melanogaster*. (B) The expression profiles for ecdysteroid synthesis and metabolism pathways during oogenesis in *D. melanogaster*. The explanation of gene identities is in the Additional file 12. (PDF 655 kb)

Additional file 9: The key factors in consensus modules between *B. mori* and *D. melanogaster*. (XLS 29 kb)

Additional file 10: The quantitative PCR primers of typical marker genes. (XLS 28 kb)

Additional file 11: The expression profiles for typical TFs and cofactors during oogenesis in *B. mori*. (A) The expression profiles for typical TFs during oogenesis in *B. mori*. (B) The expression profiles for typical cofactors during oogenesis in *B. mori*. (PDF 603 kb)

Additional file 12: The explanation of gene identities. (XLS 284 kb)

## Abbreviations

20E: 20-hydroxyecdysone
FDR: false discovery rate
FPKM: fragments per kilobase of exon model per million mapped fragments
GO: Gene Ontology
HMM: hidden Markov model
JH: juvenile hormone
KEGG: Kyoto Encyclopedia of Genes and Genomes
P7D: pupae on day 7
PCA: principal component analysis
RH-5992: 20E agonist tebufenozide
RPKM: reads per kilobase of exon model per million mapped fragments
WGCNA: weighted gene co-expression network analysis
