# Supplementary figures and images for: The dynamic landscape of gene regulation during Bombyx mori oogenesis
Source: BMC Genomics. 2017 Sep 11;18:714. doi: 10.1186/s12864-017-4123-6 (PMC5594438; doi:10.1186/s12864-017-4123-6)

*VMP30*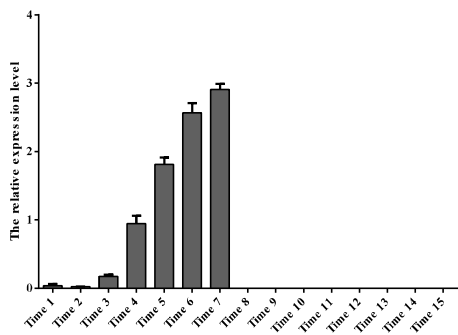*Era*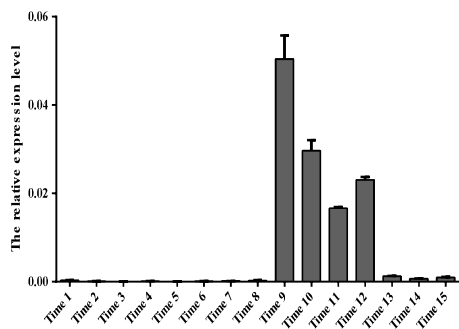*Cyp18a1*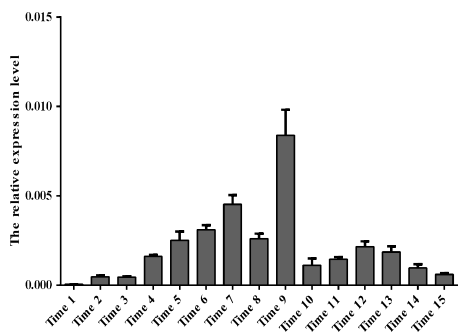*E22k*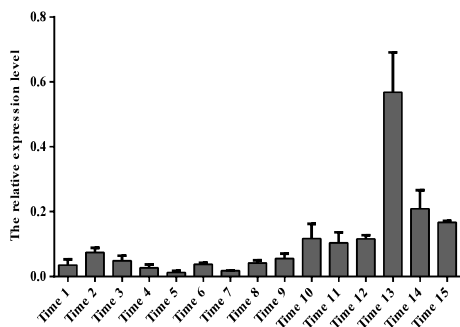*EO*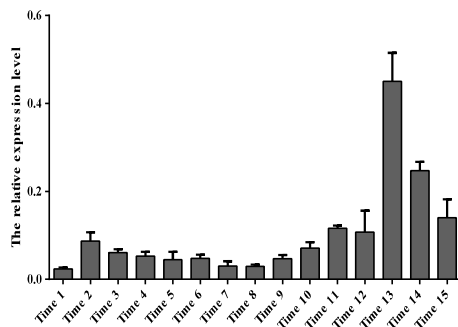*Foxo*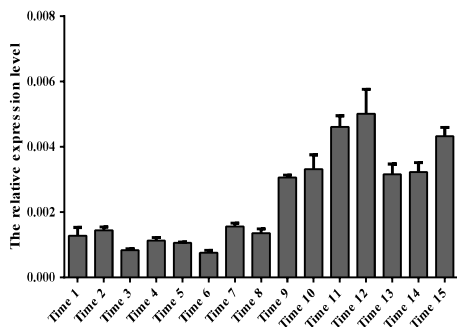*Shd*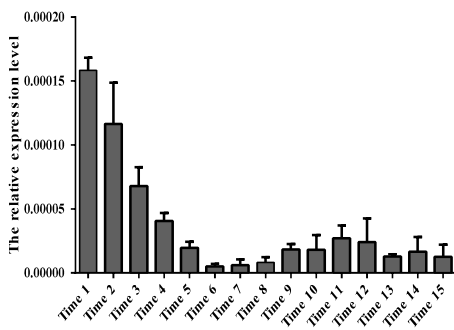*Sad*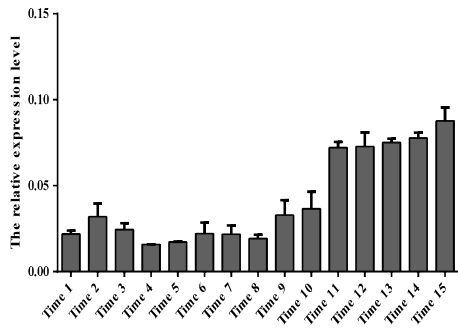

Supplement: Supplementary file 2 — The quantitative PCR expression results of typical marker genes. The explanation of gene identities is in the Additional file 12. (PDF 444 kb) [file 12864_2017_4123_MOESM2_ESM.pdf]

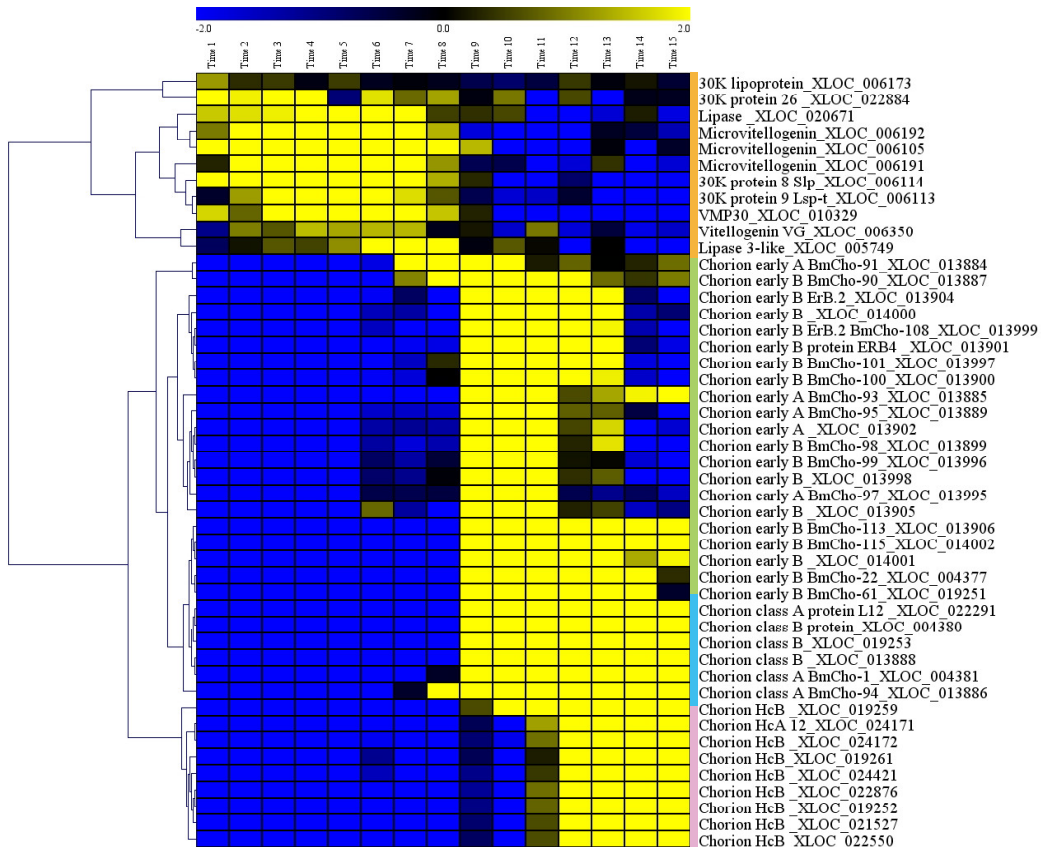

Supplement: Supplementary file 3 — The expression profiles for typical protein families during oogenesis in B. mori. (PDF 1351 kb) [file 12864_2017_4123_MOESM3_ESM.pdf]

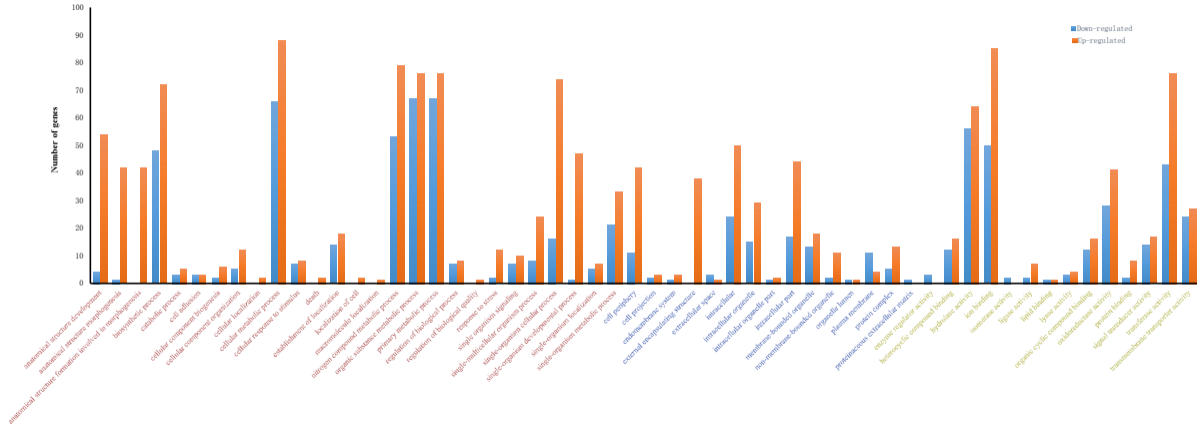

Supplement: Supplementary file 4 — The GO classifications of differentially expressed genes between Time 8 and Time 9. The up-regulated and down-regulated genes are marked in red and blue, respectively. The classifications of molecular function, cell component and biological process are represented in different colors. (PDF 600 kb) [file 12864_2017_4123_MOESM4_ESM.pdf]

A

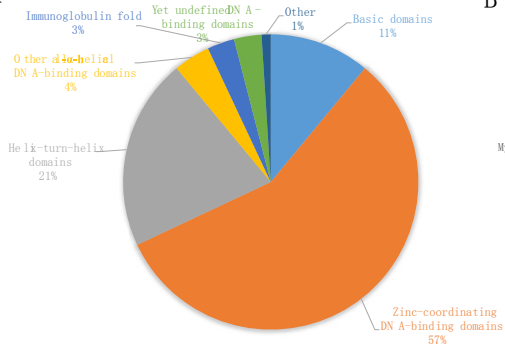

B

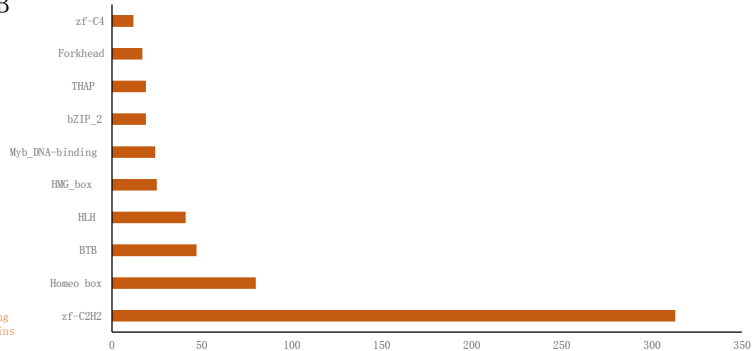

Supplement: Supplementary file 6 — The statistics of transcription factors. (A) The classification of TFs. (B) TF families with the top 10 numbers. (PDF 301 kb) [file 12864_2017_4123_MOESM6_ESM.pdf]

A

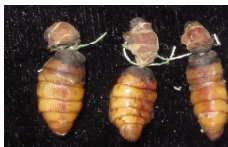

B

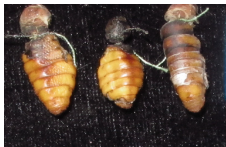

C

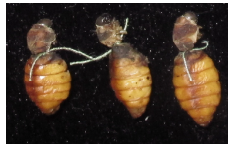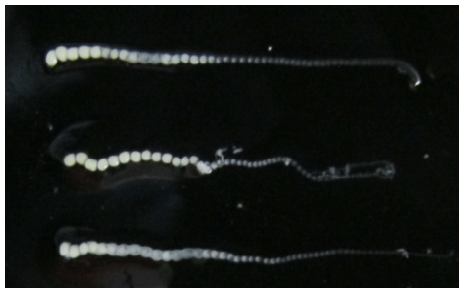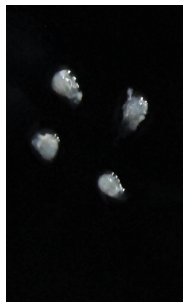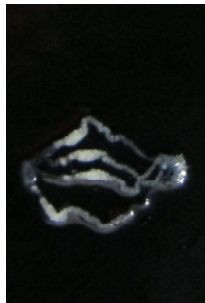

D

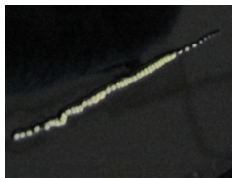

E

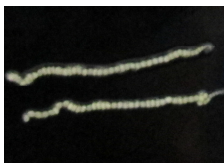

F

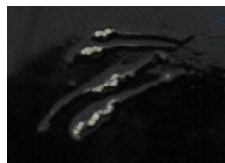

G

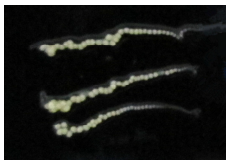

H

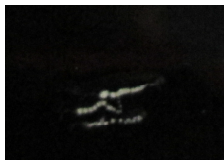

Supplement: Supplementary file 7 — The morphological changes of ovaries following hormone and inhibitor treatments. (A-C) The morphological changes observed following the 20E, 30% alcohol and RH-5992 treatments, respectively. (D-G) The morphological changes in the DMSO control and various inhibitors of insulin pathways, LY294002, U0126 and Rapamycin, respectively. (H-I) The quantitative PCR expression results of typical marker genes. The explanation of gene identities is in the Additional file 12. (PDF 14262 kb) [file 12864_2017_4123_MOESM7_ESM.pdf]

A

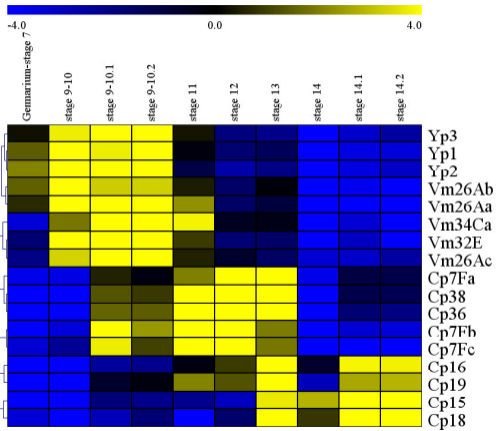

B

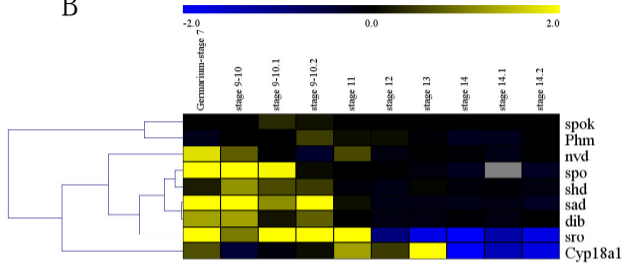

Supplement: Supplementary file 8 — The expression profiles of typical genes during oogenesis in D. melanogaster. (A) The expression profiles for typical protein families during oogenesis in D. melanogaster. (B) The expression profiles for ecdysteroid synthesis and metabolism pathways during oogenesis in D. melanogaster. The explanation of gene identities is in the Additional file 12. (PDF 655 kb) [file 12864_2017_4123_MOESM8_ESM.pdf]

A

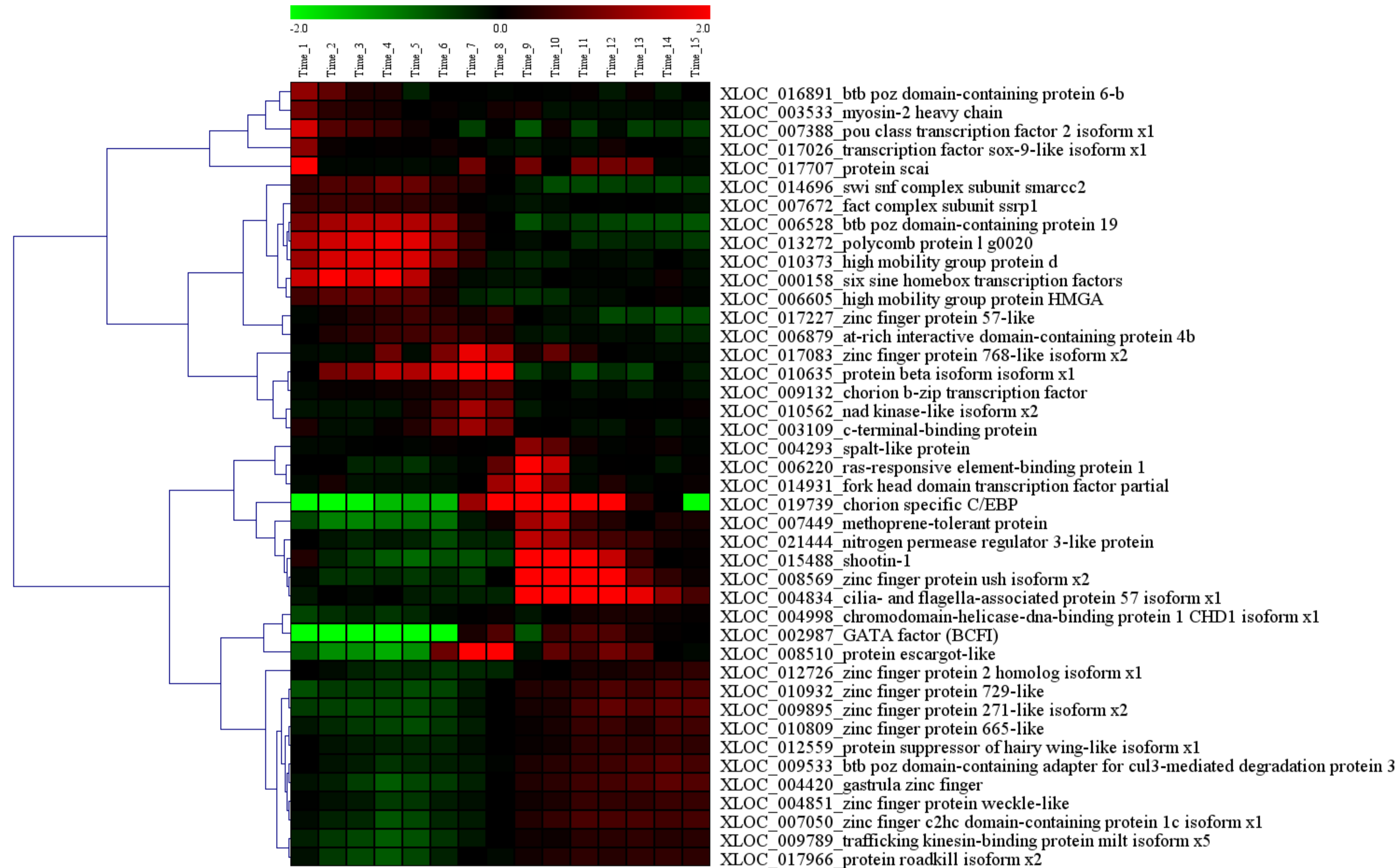

B

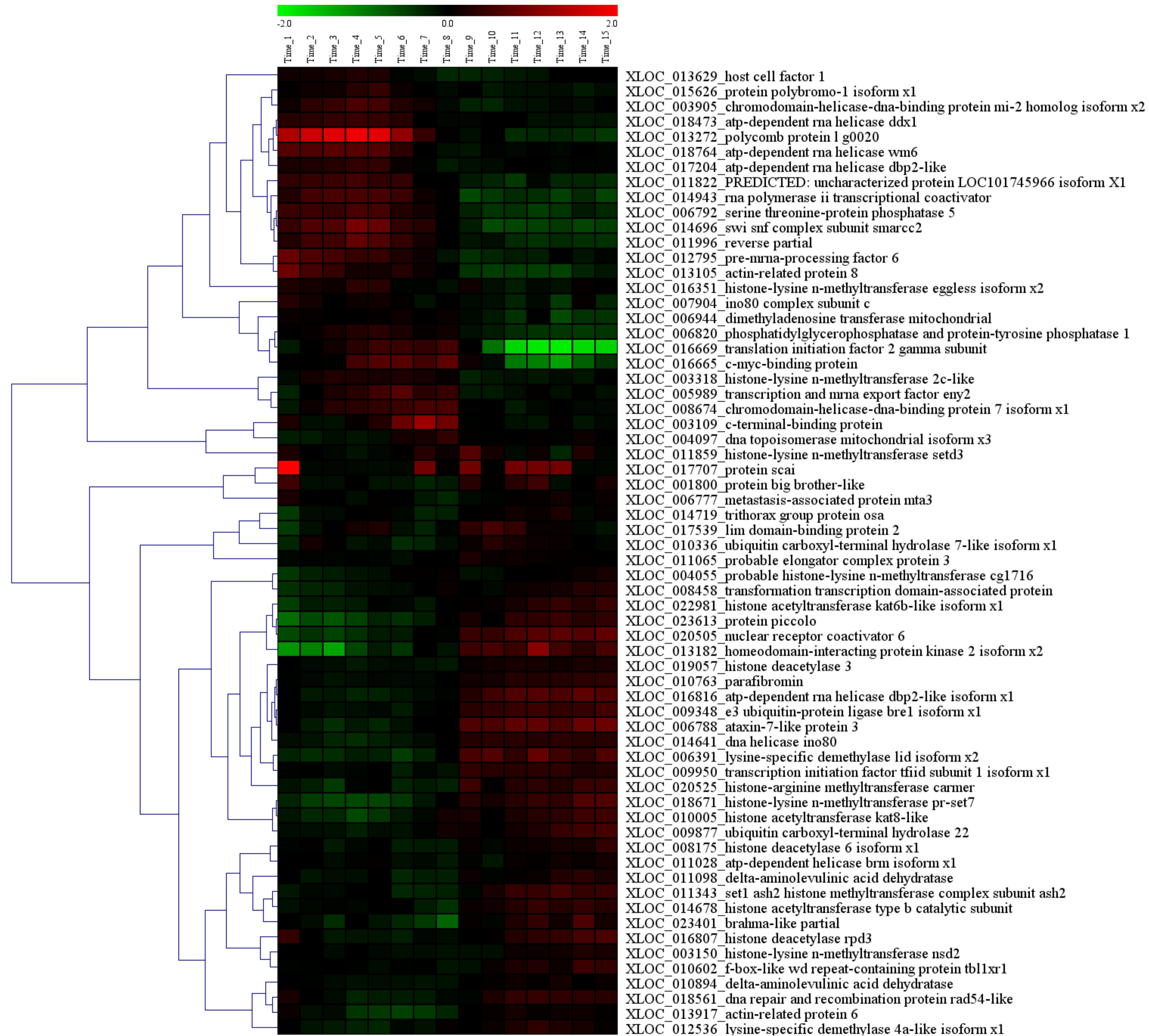

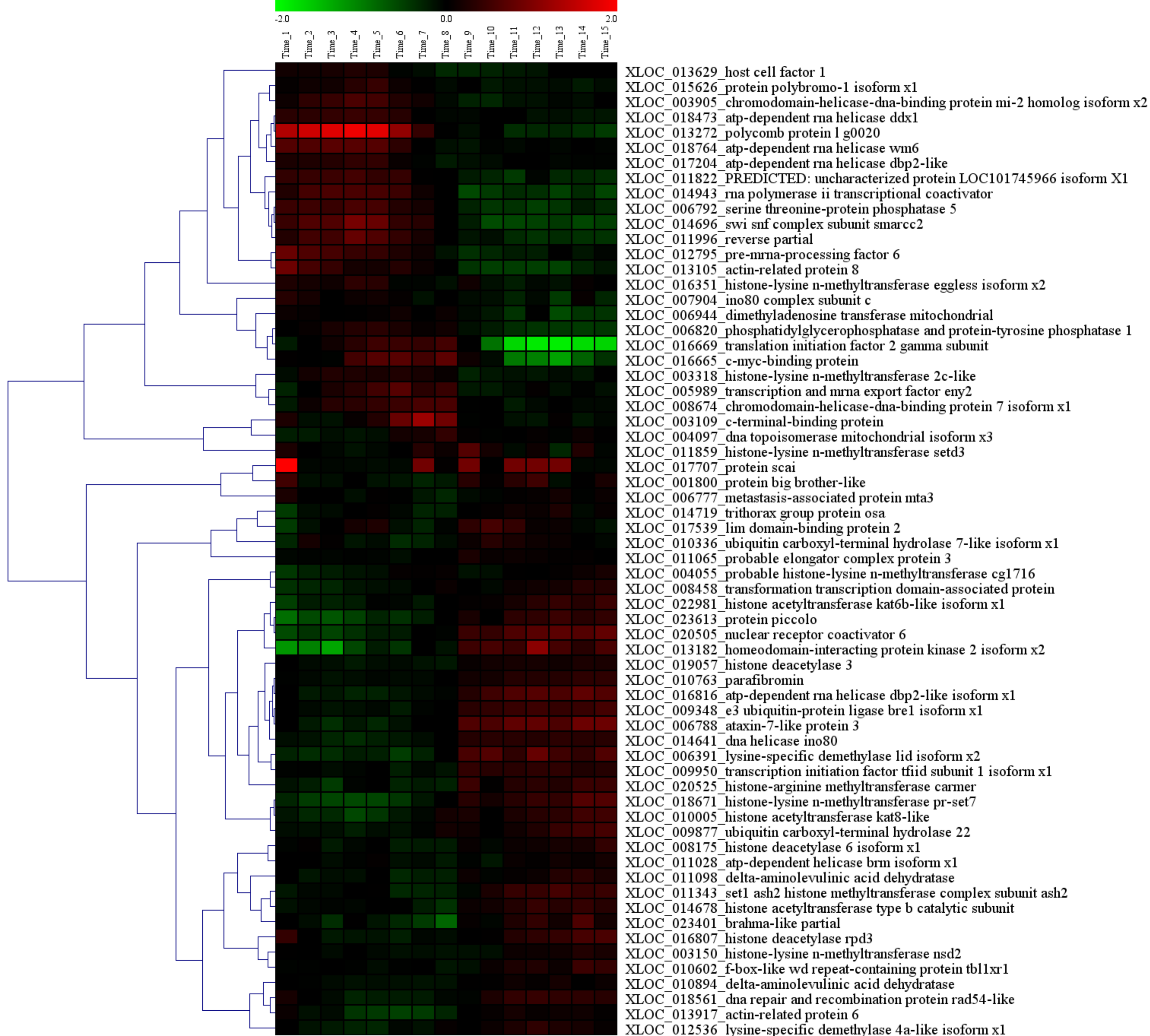

Supplement: Supplementary file 11 — The expression profiles for typical TFs and cofactors during oogenesis in B. mori. (A) The expression profiles for typical TFs during oogenesis in B. mori. (B) The expression profiles for typical cofactors during oogenesis in B. mori. (PDF 603 kb) [file 12864_2017_4123_MOESM11_ESM.pdf]
